# Regulation of the proteostasis network by the neuronal system

**DOI:** 10.3389/fmolb.2023.1290118

**Published:** 2023-11-02

**Authors:** Huadong Zhu, Ehud Cohen

**Affiliations:** Department of Biochemistry and Molecular Biology, The Institute for Medical Research Israel—Canada (IMRIC), The Hebrew University School of Medicine, Jerusalem, Israel

**Keywords:** proteotoxicity, proteostasis, neurodegeneration, neuropeptides, *C. elegans*, Alzheimer’s disease

## Abstract

The protein homeostasis (proteostasis) network is a nexus of molecular mechanisms that act in concert to maintain the integrity of the proteome and ensure proper cellular and organismal functionality. Early in life the proteostasis network efficiently preserves the functionality of the proteome, however, as the organism ages, or due to mutations or environmental insults, subsets of inherently unstable proteins misfold and form insoluble aggregates that accrue within the cell. These aberrant protein aggregates jeopardize cellular viability and, in some cases, underlie the development of devastating illnesses. Hence, the accumulation of protein aggregates activates different nodes of the proteostasis network that refold aberrantly folded polypeptides, or direct them for degradation. The proteostasis network apparently functions within the cell, however, a myriad of studies indicate that this nexus of mechanisms is regulated at the organismal level by signaling pathways. It was also discovered that the proteostasis network differentially responds to dissimilar proteotoxic insults by tailoring its response according to the specific challenge that cells encounter. In this mini-review, we delineate the proteostasis-regulating neuronal mechanisms, describe the indications that the proteostasis network differentially responds to distinct proteotoxic challenges, and highlight possible future clinical prospects of these insights.

## The integrity of the proteome is supervised and maintained by the proteostasis network

The maintenance of cellular and organismal functionality requires a tight supervision of protein integrity throughout the lifecycles of these molecules, from synthesis to degradation. Specialized, highly conserved mechanisms, maintain and coordinate the integrity of the proteome across the organism to promote protein homeostasis (proteostasis). This collection of mechanisms, known as the “proteostasis network”, act in a coordinated manner to preserve the functionality of proteins by various activities. These activities include, assisting nascent polypeptide attaining their accurate spatial structures, ensuring the addition of proper post-translational modifications, supervising protein-protein interactions and directing terminally misfolded proteins for degradation (Jayaraj et al., 2019). Nevertheless, aging, mutations and environmental stressors, suppress the efficiency of the proteostasis network, enabling subsets of aggregation-prone proteins escaping the cellular surveillance mechanisms and form insoluble aggregates that accumulate within the cell. This hazardous process underlies the development of a myriad of diseases that were collectively termed “proteinopathies” (Paulson, 1999). Late onset neurodegenerative disorders such as Alzheimer’s (AD), Parkinson’s (PD), Huntington’s diseases (HD) and Amyotrophic Lateral Sclerosis (ALS) are relatively prevalent, incurable proteinopathies that set a major burden on public health systems throughout the industrialized world. Therefore, understanding how proteostasis is regulated and orchestrated at the cellular and organismal levels is of great importance as these insights bear the promise to enable clinical interventions which will harness the capabilities of this network to delay, and perhaps even prevent, the manifestation of various devastating late-onset diseases. Since the links between proteostasis and aging (Hipp et al., 2019; Morimoto, 2020) and those which associate proteostasis collapse and neurodegeneration (Hoppe and Cohen, 2020) have been reviewed recently, here we will focus on delineating the signaling mechanisms that orchestrate proteostasis across tissues.

## Signals of multiple pathways integrate to regulate proteostasis across the organism

The apparent cell autonomous nature of proteostasis-modulating mechanisms and the ability of unicellular organisms and of cultured cells to respond to acute proteostasis impairments, such as heat stress (Lindquist, 1986), have led researchers to assume that proteostasis is orchestrated at the cellular level. However, this notion has been scrutinized and challenged by a series of studies that were mostly conducted using the nematode *C. elegans* (*Caenorhabditis elegans*). The investigation of proteostasis across tissues requires a model organism that enables the manipulation of signaling in a specific tissue and monitoring the toxicity of protein aggregation (proteotoxicity) in other tissues. The nematode offers great advantages for the study of proteostasis due to several features (Volovik et al., 2014a). It has a short and well-defined lifecycle that enables the study of proteostasis in the context of aging. RNA interference (RNAi) enables highly efficient and specific gene knockdown across the organism or in a tissue specific manner. A variety of proteotoxicity-model strains are obtainable (Caldwell et al., 2020) and the nematodes are transparent. This unique feature, which allows the visualization of fluorescent proteins, enables a concurrent tracking of gene expression and protein aggregation in different tissues of a living nematode (Volovik et al., 2014a).

One example of inter-tissue communication is the connection between the AFD neurons, their neighboring AIY interneurons, and distal tissues. The AFD neurons are known to be crucial for heat sensing and avoidance, a behavior that was termed “thermotaxis” (Mori and Ohshima 1995). Thermotaxis is entirely dependent on the guanylyl cyclase GCY-8 in these neurons (Inada et al., 2006). The activity of this neural mechanism was reported to be crucial also for the activation of the “heat shock response” (HSR), a stress response mechanism that is acted upon the accumulation of misfolded proteins in the cytosol of cells that were exposed to elevated temperatures (Prahlad et al., 2008). This study indicates that the HSR, which functions to restore proteostasis, is regulated at the organismal level by neuron-to-soma signaling. A follow up study has shown that this neuronal mechanism also modulates proteotoxicity in distal tissues of worms that are challenged by chronic, neurodegeneration-associated proteotoxic challenge (Prahlad and Morimoto, 2011).

These seminal findings raised the prospect that additional neuronal components are involved in proteostasis regulation across tissues. Searching for neuronal receptors that are needed for HSR activation we discovered that *gtr-1*, a gene which encodes a neuronal G-protein-coupled receptor (GPCR), is needed for HSR activation in non-neuronal tissues (Maman et al., 2013). Surprisingly, *gtr-1* is expressed in chemosensory neurons indicating that these cells are also involved in HSR regulation in the soma. In addition, the ubiquitin transferase NHL-1 which resides in chemosensory neurons, is an HSR regulator which differentially controls the activation of the proteostasis-promoting transcription factors DAF-16/FOXO and HSF-1 (Volovik et al., 2014b). These transcription factors, as well as SKN-1/NRF and PQM-1, are regulated by key aging regulating pathways including the insulin/IGF signaling (IIS) cascade, a prominent aging-controlling pathway in worms (Kenyon et al., 1993) and mammals (Holzenberger et al., 2003).

Yet, the regulation of protein quality-control mechanisms by neurons is not limited to the HSR. An additional stress response signaling pathway that governs proteostasis by neuronal signaling is the “unfolded protein response” (UPR) of the endoplasmic reticulum (UPR^ER^). This link was uncovered by the observation that the expression of a constitutively active isoform of the transcription factor XBP (XBPs) in neurons, activates the UPR^ER^ in remote tissues (Taylor and Dillin, 2013).

Neurons are not the only cells that signal to other tissues to confer proteostasis. Signals that originate from the reproductive system govern both acute proteotoxicity inflicted by heat stress (Shemesh et al., 2013; Labbadia and Morimoto, 2015) and chronic proteostasis impairment by the expression of neurodegeneration-linked proteins (Moll et al., 2018). Interestingly, *gnrr-2* which codes a neuronal GPCR, is a proteostasis regulator that functions downstream of signaling that originates from the reproductive system, and regulates the transcription factors DAF-16/FOXO, HSF-1 and PQM-1 (Kishner et al., 2022). In fact, signals that emanate from the reproductive system as a result of DNA damage, integrate with signals that stem from the intestine, an emerging proteostasis-coordinating tissue (Hodge et al., 2022), due to exposure to pathogens to enhance UPS activity and promote proteostasis across the organism (Ermolaeva et al., 2013). Likewise, one of the proteostasis-controlling mechanisms downstream of the IIS involves the regulation of GLP-1, a lifespan regulating (H et al., 1999; Arantes-Oliveira et al., 2002) component of the Notch receptor that resides in the worm’s germline, to regulate proteostasis in other tissues. The IIS regulates GLP-1 activity by reducing the SUMOylation of the regulatory protein CAR-1 thereby suppressing GLP-1 activity (Moll et al., 2018).

The important roles of the reproductive system in proteostasis coordination across tissues were also demonstrated by the aggregation of PGL-1 in the mitochondria of germ cells. This aggregation leads to the activation of the UPR of the mitochondria (UPR^mito^), to mitochondrial fragmentation and to proteostasis impairments in distal tissues including neurons, muscles and the intestine. This regulation is dependent on the activity of Wnt signaling (Calculli et al., 2021).

Another inter-tissue, proteostasis-promoting mechanism is the transcellular chaperone signaling (TCS) which promotes communication between intestinal and muscle cells upon the expression of misfolding-prone proteins. This communication mechanism, which leads to elevated expression levels of the heat shock protein 90 (HSP90) in muscle, intestinal and pharyngeal cells, is dependent upon the transcription factor PQM-1 (O'Brien et al., 2018) and in a subset of TCS-activating genes (Miles et al., 2023).

Collectively, these studies culminate to substantiate the theme that proteostasis is controlled, at least partially, at the organismal level by various signals that originate from different organs and integrate to regulate the integrity of the proteome across tissues in a coordinated manner. They also show that a small subset of proteostasis-promoting transcription factors serve as functional junctions, as they assimilate these signals to regulate proteostasis in different tissues. These insights raise the key question of whether the proteostasis network similarly responds to acute and chronic proteotoxic insults.

## The ability to respond to heat can come at the expense of mitigating chronic proteotoxicity

Since both, exposure to an elevated temperature and a constant expression of proteotoxic proteins, lead to proteostasis impairments, it was conceivable that the knockdown of *gtr-1, gcy-8* or of *nhl-1* would enhance proteotoxicity that stems from the aggregation of neurodegeneration-causing proteins. However, surprisingly this is not the case. Using worms that express the AD-causing, aggregation-prone Aβ_3-42_ peptide in their muscles, expression that leads to a progressive paralysis within the worm population (Link, 1995), we found that while the knockdown of *gtr-1* or of *nhl-1* by RNAi abolishes the worms’ ability to activate the HSR, it mitigates Aβ-mediated proteotoxicity in these animals (Maman et al., 2013; Volovik et al., 2014b). In agreement, the knockdown of the *cav-1* gene, that encodes a protein that is essential for the formation of neuronal caveolae, a subtype of membrane microdomains (Parton and Simons, 2007), protects CL2006 worms from proteotoxicity, but does not modulate heat stress resistance (Roitenberg et al., 2018). This work proposes that caveolae serve as a scaffold for the assembly of neuronal signaling complexes that regulate proteostasis in the soma. Intriguingly, the protection from Aβ toxicity that was observed upon the knockdown of *cav-1* was HSF-1-dependent, but independent in the activities of DAF-16/FOXO (Cohen et al., 2006) and SKN-1/NRF (Blackwell et al., 2015) adding an additional indication that these proteostasis-promoting transcription factors are differentially regulated.

**FIGURE 1:**
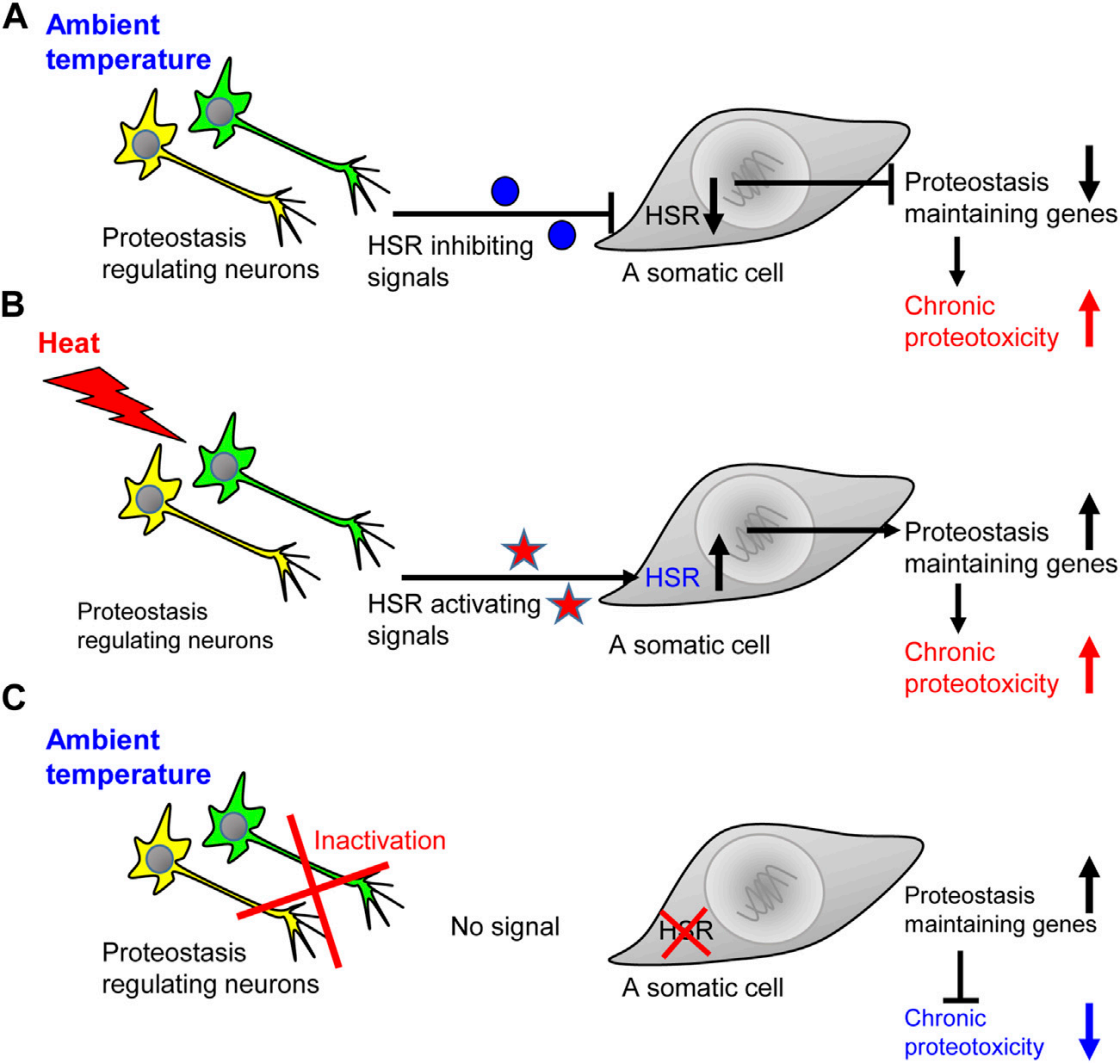
The ability to activate the heat shock response comes at the expense of coping with chronic proteotoxicity. **(A)** When the worm is in the ambient temperature, the network of HSR-regulating neurons send inhibitory signals that prevent the expression of genes that code for key folding chaperones. This inhibition exposes the worm to the toxicity of aggregation-prone proteins and impair proteostasis, thereby jeopardizing cellular and organismal functionality. **(B)** An exposure to elevated temperature leads to the accumulation of misfolded proteins and initiates the secretion of HSR-activating signals which induce the expression of folding chaperones and other proteostasis promoting proteins. Nevertheless, in the face of the acute proteostasis collapse induced by heat, chronic proteotoxicity is also enhanced. **(C)** When the worm is in the ambient temperature and its HSR-regulating neuronal network is inactivated, somatic cells do not receive inhibitory signals. Thus, the cells can more efficiently respond to chronic proteotoxicity by elevating the expression levels of proteostasis-promoting genes.

Similarly, the knockdown of *gcy-8* in worms that express proteotoxic, abnormally long glutamine stretches, known to cause neurodegeneration (Paulson et al., 2000), protects from proteotoxicity (Prahlad and Morimoto, 2011). These unexpected observations suggested that neurons not only activate the HSR upon exposure to elevated temperatures, but also send negative signals that suppress the induction of proteostasis-promoting chaperones when the worm is in the ambient temperature. Indeed, the knockdown of *gcy-8* by RNAi results in a more prominent induction of the heat shock protein 70 (Hsp70) in worms that express polyQ-YFP stretches compared to nematodes of the same strain that were grown on control bacteria and thus, uninterruptedly expressed *gcy-8* (Prahlad and Morimoto, 2011). Accordingly, it is clear that neurons regulate the organismal responses to acute and chronic proteotoxic insults. However, it is also apparent that the ability to respond to acute stress can come at the expense of the organismal ability to cope with chronic proteotoxic insults (Figure 1). These insights raise key questions including: What are the messengers that carry the different proteostasis-promoting signals to the soma, and whether the proteostasis network specifically tailors its responses according to the proteotoxic insult that challenges the organism?

## Neurotransmitters and neuropeptides carry the signals that orchestrate proteostasis in distal tissues

Using worms that express fluorescently-tagged HSF-1 the Prahlad laboratory has shown that the induction of serotonin secretion by experimental means, drives HSF-1 into cell nuclei in remote tissues (Tatum et al., 2015). This observation nominated this neurotransmitter as one of the neuronal messengers that activate the HSR in remote tissues. This notion was supported by the finding that serotonin also mitigates chronic proteotoxicity in worms and mice. The application of citalopram, a selective serotonin reuptake inhibitor (SSRI) (that elevates the levels of serotonin), protects model worms and mice that were engineered to express a proteotoxic Ataxin 3 mutant which bears abnormally long polyQ stretches (Teixeira-Castro et al., 2015). This proteotoxic Ataxin 3 underlies the manifestation of the neurodegenerative disorder “Machado Joseph Disease” (MJD) in humans (Durcan and Fon, 2013). Fluoxetine, another drug of the SSRI class, mitigates proteotoxicity that is conferred by Aβ in worms (Keowkase et al., 2010) and protects middle-aged AD model mice from loss of dendritic spines (Ma et al., 2020). Together, these studies confirm that the key roles of serotonin as an important regulator of proteostasis are conserved from worms to mammals.

Are neuropeptides also involved in the orchestration of proteostasis across the organism? In fact, neuropeptide signaling that stems from glial cells was reported to regulate the UPR^ER^ and lifespan of *C. elegans* (Frakes et al., 2020). However, do neuropeptides also govern the response to chronic proteotoxicity? And if yes, do they similarly respond to distinct proteotoxic insults? To address these questions, we employed different proteotoxicity worm models, and asked whether chaperones that are known to mitigate polyQ-mediated proteotoxicity, necessarily protect animals from Aβ aggregation. The rationale in the basis of this approach suggested that if the proteostasis network differentially responds to distinct proteotoxic proteins, certain components of this network may be proved protective when a certain proteotoxic protein is expressed but deleterious in the face of another proteotoxic challenge. Using the paralysis assay to measure the proteotoxicity of Aβ_3-42_ and the thrashing assay to follow the toxicity of polyQ35 fused to the yellow fluorescent protein (polyQ35-YFP)) we discovered that *tor-1* and *tor-2*, chaperone-encoding genes that were reported to protect nematodes from the aggregation of polyQ (Caldwell et al., 2003), enhances Aβ proteotoxicity. Similar results were obtained when the same proteotoxic proteins were expressed in muscles and neurons (Boocholez et al., 2022). To explore the mechanism that differentially regulates proteostasis in remote tissues we conducted an RNA-Seq experiment, asking which genes respond in opposition to the knockdown of *tor-1* and *tor-2* in animals that express Aβ_3-42_ and in their counterparts that express polyQ35-YFP. Among other genes we identified a subset of three neuropeptide-encoding genes: *nlp-13, nlp-18, nlp-49* and the gene *dpsm-1*. The knockdown of each of these neuropeptides encoding genes as well as of *dpsm-1,* protects from Aβ proteotoxicity but exacerbates the toxicity of polyQ35-YFP. SKN-1 was surprisingly found to be deleterious when *tor-1* and *tor-2* are knocked down by RNAi in worms that face polyQ35-YFP toxicity but protective in worms that express Aβ and were treated with the same RNAi.

**FIGURE 2:**
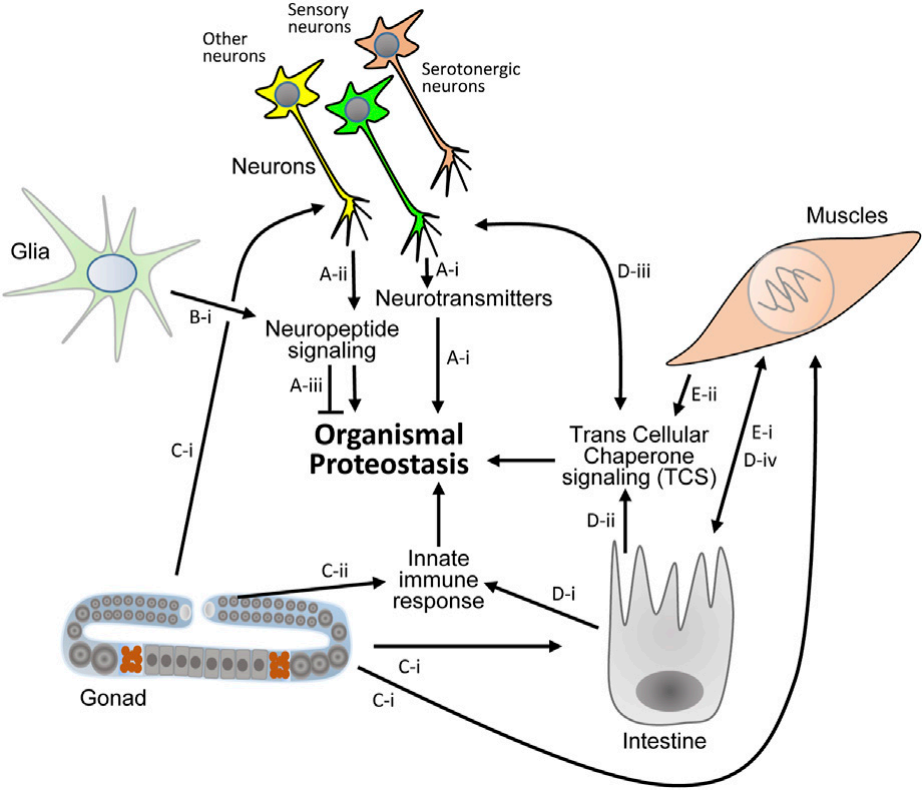
Known proteostasis-promoting signaling mechanisms in the nematode. **(A)** Thermosensation has been reported to activate serotonergic neurons and initiate the secretion of serotonin which controls proteostasis in the soma (**(A)**-I, Ref. 9, 29). Sensory and other neurons secret neuropeptides to differentially activate responses of the proteostasis network in distal cells (**(A)**-ii, Ref. 36). Specific neuropeptide signaling can impair proteostasis that stems from certain proteotoxic proteins (**(A)**-iii, Ref. 36). **(B).** Proteostasis promoting neuropeptide signaling is also activated by glial cells (**(B)**-i, Ref. 34). **(C–E)** Protein aggregation in mitochondria of germ cells signals to neurons, intestine and muscle cells to orchestrate the organismal response to proteostasis impairments (**(C)**-i, Ref. 19). Immune response activating signals that stem from the germline collaborate with intestinal signaling that is activated upon exposure to pathogens to activate the UPS and promote proteostasis across tissues (**(C)**-ii, **(D)**-i Ref. 18). The intestine is also pivotal for TCS activation (**(D)**-ii) that signals to neurons (**(D)**-iii) and to muscles (**(D)**-iv, Ref. 20). Muscle cells communicate with the intestine (**(E)**-i) and activate TCS (**(E)**-ii) via a subset of transcellular-X-cross-tissue (txt) genes and transcription factors (**(E)**-I, Ref. 21).

These discoveries clearly show that certain neuropeptides and neurotransmitters are proteostasis modulators. They also indicate that certain neuropeptides and downstream transcription factors can play opposing roles when distinct proteotoxic proteins challenge the organism (Figure 2).

Since the investigation of the roles of these molecules as regulators of aging and proteotoxicity is in its infancy, we expect that many additional neuronal, proteostasis-controlling messengers will be discovered in the foreseen future. Nonetheless, the current knowledge clearly indicate that these molecules may have clinical relevance in future treatments for neurodegenerative maladies.

## Potential clinical implications

Despite major efforts and vast financial investments, nearly all clinical trials that were designed based on the amyloid hypothesis, have failed (Panza et al., 2019). Even the antibody lecanemab, which has been recently approved for the treatment of AD, is not necessarily a breakthrough for the treatment of AD, as its efficacy and safety require further clarification (Couzin-Frankel, 2023). This grim situation requires rethinking of how neurodegenerative disorders in general and AD in particular, could be treated and managed. The understanding that aging-regulating pathways are involved in exposing the elderly to neurodegeneration (Cohen et al., 2009; Gontier et al., 2015), and the indications that brain atrophy occurs long before clinical dementia becomes evident (Fox et al., 1999), underscored the need for early diagnosis and preventive intervention. In fact, studies that were focused on the “Colombian cohort”, an extended family with many of its members inherent an AD-causing mutation, indicated that massive amyloid deposition is evident 15 years before early clinical signs of dementia. Brain volume loss can be also observed a decade or more, prior to the diagnosis of mild cognitive impairment (Fuller et al., 2019). This long incubation period provides a wide window of opportunities for preventive interventions and highlights the importance of the development of diagnostic tools for early detection of AD. Despite the great importance of early diagnosis, we will not discuss this aspect here but focus on the therapeutic potential of the promotion of organismal proteostasis as an early preventive intervention.

Since AD and other neurodegenerative disorders are multi-factorial syndromes, it is plausible that future AD treatments will be combinatorial. Such cocktails will be designed to concurrently modify the activities of different biological mechanisms. However, what drugs such therapeutic cocktails should be consist of? Since aging plays critical roles in exposing elder model animals to neurodegeneration (Cohen et al., 2009; Gontier et al., 2015), aging-modulating agents are likely to be important components of future counter-proteotoxic therapeutic cocktails. One such compound may be highly efficient IGF1 signaling inhibitors like NT219 that was shown to protect worms from proteotoxicity (El-Ami et al., 2014) and to induce the expression of key chaperones in mammalian cells (Moll et al., 2016). Reducing the rate of neuro-inflammation is also beneficial in maintaining brain functionality over time. Thus, anti-inflammatory drugs should be also considered.

The studies that we described herein point at certain neurotransmitters and neuropeptides as additional promising proteostasis modulators. Hence, elevating the levels of serotonin by SSRIs and a periodic infusion of certain neuropeptides, may be also contained in future therapies. Nevertheless, the discovery that components of the proteostasis network can play opposing roles in the face of different proteotoxic challenges, highlights the importance of careful and specific tailoring of therapeutic cocktails of neuropeptides and/or neurotransmitters according to the subtype of disease that the individual patient is expected to develop or already suffers from.

In sum, early diagnosis, careful characterization and classification of disease subtypes as well as the development of disease-specific combinatorial therapeutic cocktails hold the promise to set a new era in our ability to delay the onset, slow the progression of neurodegeneration and provide the elder patients with additional healthy and productive years.

## Abbreviations

AD, Alzheimer’s disease; ALS, Amyotrophic Lateral Sclerosis; Aβ, Amyloid β peptide; ER, endoplasmic reticulum; GPCR, G protein coupled receptor; HD, Huntington’s disease; HSR, heat shock response; IGF, Insulin-like growth factor; IIS, Insulin/IGF signaling; MJD, Machado Joseph Disease; PD, Parkinson’s disease; PolyQ, poly-glutamine; Proteostasis, protein homeostasis; Proteotoxicity, toxic protein aggregation; RNAi, RNA interference; TCS, transcellular chaperone signaling; UPR, unfolded protein response; YFP, yellow fluorescent protein.
